# KREO Prevents CUMS‐Induced Depressive‐Like Behavior via Modulation of the BDNF Signaling Pathway

**DOI:** 10.1002/fsn3.71912

**Published:** 2026-05-27

**Authors:** Yanni Wang, Zhaorong Yue, Manjie Xu, Wenjun Xu, Weizhong Zhang, Xiaolong Li, Qinghong Liu, Kailong Guan, Zewen Zhang, Yang Li, Xin Wang, Hongyu Li

**Affiliations:** ^1^ Institute of Microbiology, School of Life Sciences Lanzhou University Lanzhou China; ^2^ School of Pharmacy Lanzhou University Lanzhou China

**Keywords:** 5‐HT, anti‐depression, BDNF signaling pathway, Chinese Kushui rose essential oil, corticosterone

## Abstract

Chinese Kushui Rose (*Rosa sertata × Rose rugosa*) is widely recognized for its medicinal and edible properties, and Chinese Kushui Rose Essential Oil (KREO) has attracted growing attention due to its potential anxiolytic and sedative‐hypnotic effects. This study aims to systematically evaluate the preventive effects of KREO against depression‐like behaviors and explore its underlying mechanisms. We identified 57 volatile compounds from KREO using gas chromatography–mass spectrometry (GC–MS), with the major constituents being citronellol, geraniol, and citronellyl acetate. In mice with chronic unpredictable mild stress (CUMS)‐induced depression, administration of KREO facilitated body weight recovery, alleviated depressive‐like behaviors, and improved voluntary locomotor activity and exploratory behavior. Mechanistic investigations revealed that KREO increased hippocampal levels of serotonin (5‐HT) and brain‐derived neurotrophic factor (BDNF), reduced serum corticosterone (Cort) concentrations, and upregulated the expression of tyrosine receptor kinase B (TrkB) as well as the phosphorylation of extracellular signal‐regulated kinase 1/2 (p‐ERK1/2) in the hippocampus. Thus, KREO prevents depression‐like behavior by enhancing monoaminergic neurotransmission and attenuating hypothalamic–pituitary–adrenal (HPA) axis hyperactivity, with these effects associated with activation of the BDNF signaling pathway. These findings support the potential of KREO as a therapeutic intervention for depression and as a functional dietary ingredient.

## Graphical abstract

This study showed that Chinese Kushui Rose Essential Oil (KREO) can alleviate depressive symptoms, increase voluntary activity, and enhance exploratory behavior in mice induced with chronic unpredictable mild stress (CUMS) by elevating serotonin (5‐HT) levels and reducing hyperactivity of the hypothalamic–pituitary–adrenal (HPA) axis. Notably, KREO elevated brain‐derived neurotrophic factor (BDNF) levels, promoted tyrosine receptor kinase B (TrkB) expression, and induced extracellular signal‐regulated kinase 1/2 (ERK1/2) phosphorylation in the hippocampus.

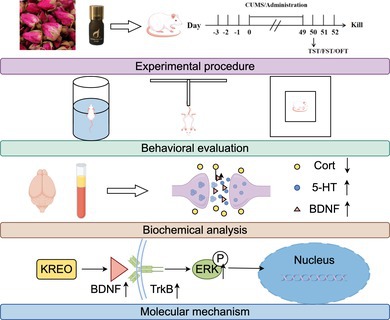

## 1 Introduction

Depression is a chronic and recurrent condition that persists throughout an individual's lifetime, with nearly 80% of patients experiencing at least one recurrence episode (Malhi and Mann 2018). It is primarily characterized by persistent sadness and anhedonia. As the disease progresses, individuals may develop feelings of shame and worthlessness, which can ultimately lead to suicidal ideation (McCarron et al. 2021). According to the China Mental Health Survey, the lifetime prevalence of depression among adults is approximately 6.8% (Huang et al. 2019). In addition to its high prevalence and recurrence rates, the complex pathogenesis of depression presents a significant challenge in its clinical management.

The hippocampus plays a central role in the investigation of the underlying mechanisms of depression, as it is interconnected with various brain regions involved in mood regulation and contributes to emotional processing (Cullen et al. 2014; Hao et al. 2020). Hippocampal volume reduction is one of the most extensively documented neuroanatomical abnormalities associated with depression (Belleau et al. 2019). 5‐HT plays a critical role in regulating hippocampal neurogenesis, maintaining normal hippocampal function, and modulating emotional information processing in the brain (Kraus et al. 2017). Chronic stress can elevate plasma cortisol levels, a hallmark feature of depression (Swaab et al. 2005). Elevated cortisol disrupts the balance of monoamine neurotransmitter systems and reduces 5‐HT concentrations. 5‐HT dysfunction increases an individual's vulnerability to chronic stress, thereby creating a feedback loop that exacerbates structural and functional impairments in the hippocampus, ultimately contributing to the onset or worsening of depressive symptoms (Mahar et al. 2014). The majority of currently prescribed antidepressants in clinical practice target monoaminergic systems (McCarron et al. 2021). However, these small‐molecule agents have limited efficacy in addressing the multifaceted pathophysiology of depression and are often associated with notable side effects, including insomnia, anxiety, and sexual dysfunction (Cuijpers et al. 2014). Consequently, dietary interventions are increasingly recognized as promising, safe, natural, and cost‐effective approaches with potential benefits for stress resilience and mental health.

Herbal essential oils exhibit a broad range of physiological activities and play a significant role in the food industry. Notably, they have gained increasing recognition for their potential to alleviate depressive symptoms and promote emotional well‐being (Ulrich‐Merzenich et al. 2010). Accumulating evidence highlights the therapeutic potential of various essential oils in modulating mood through multiple mechanisms. For example, lavender essential oil is recognized for its potent antidepressant effects and is approved in Germany as a treatment for anxiety and depression (Müller et al. 2021). Studies have shown that garlic essential oil exerts antidepressant‐like effects by suppressing inflammatory responses and improving gut health (Huang et al. 2023). Navel orange essential oil, particularly its primary constituent limonene, has been demonstrated to produce antidepressant effects via modulation of neuroendocrine, neurotransmitter, and neurotrophic systems (Zhang et al. 2019). Additionally, essential oils derived from *Perillae folium* and agarwood have exhibited antidepressant properties (Nguyen et al. 2024; Wang et al. 2018). Chinese Kushui Rose (*Rosa sertata × Rose rugosa*) is a cultivar native to northwest China, valued for its edible properties. KREO, a bioactive compound extracted from Chinese Kushui Rose, demonstrates notable neuroprotective and mood‐regulating effects. Previous studies have demonstrated that KREO administration prolongs sleep duration in mice (Luo et al. 2018) and significantly reduce anxiety‐like behaviors (Ding et al. 2019). Our previous research has also demonstrated that KREO exerts neuroprotective effects by inhibiting the abnormal accumulation of amyloid‐β (Aβ) and alpha‐synuclein (α‐Syn) (Muhammad et al. 2023; Zhu et al. 2017). The dual medicinal and nutritional properties of KREO make it a promising candidate for research in food science, healthcare, and pharmaceutical development. Therefore, the integration of KREO's therapeutic benefits with its culinary applications not only supports the development of novel pharmaceutical agents but also aligns with the growing interest in harnessing natural resources for health promotion, thereby attracting considerable scientific and industrial attention.

In this study, we employed a CUMS mouse model to evaluate the ability of KREO to prevent the onset of CUMS‐induced depressive‐like behavior. Levels of monoaminergic neurotransmission were assessed using enzyme‐linked immunosorbent assay (ELISA), and the potential modulation of the BDNF signaling pathway through modulation of the HPA axis was examined by western blot analysis (WB). These findings provide a theoretical foundation for incorporating herbal essential oils into dietary interventions for the treatment of depression.

## 2 Materials and Methods

### 2.1 Availability of KREO

KREO was provided by Gansu Oriental Tianrun Rose Industry Co. Ltd. (Gansu, China) and was extracted from fresh flowers of Chinese Kushui Rose using a standard steam distillation method in accordance with the Chinese national standard.

### 2.2 GC–MS Analysis and Quantification

The chemical composition of KREO was analyzed using a Trace 1300‐ISQ GC–MS system (Thermo Fisher Scientific, USA) equipped with a TG‐5MS fused silica capillary column (30 m × 0.25 mm i.d., 0.25 μm film thickness; Thermo Fisher Scientific, USA). The analytical conditions were adapted from previously described protocols (Zhu et al. 2017). The oven temperature program was initiated at 40°C and held for 3 min, then increased to 100°C at a rate of 8°C/min, subsequently to 200°C at 5°C/min, and finally to 250°C at 10°C/min, where it was maintained for 10 min. The injection port and ion source temperatures were set at 200°C and 250°C, respectively. Helium was used as the carrier gas at a constant flow rate of 1.2 mL/min. KREO was diluted 1:500 in n‐hexan (H811147, Macklin), and 1 μL of the solution was injected in split mode with a split ratio of 1:10. Mass detection was performed using an electron ionization system operated at 70 eV, with mass spectra acquired over a range of 35–600 m/z.

For qualitative analysis, compounds were tentatively identified by comparing their mass spectra with the NIST 20 database (similarity index ≥ 800) and further confirmed by matching retention times with authentic reference standards when available. Given the availability of analytical standards and their documented biological activities, six key compounds were selected for focused analysis: linalool (HY‐N0368, MCE), citronellol (HY‐W010201, MCE), geraniol (HY‐N6952, MCE), citronellyl acetate (HY‐N7144A, MCE), methyleugenol (HY‐N6996, MCE), and 2‐tridecanone (HY‐W009811, MCE).

Quantitative analysis was performed using an internal standard‐corrected external calibration method. A calibration series consisting of 5–7 concentration levels for each target analyte was prepared in matrix‐matched solvent and analyzed under strictly identical chromatographic conditions. n‐Tetradecane (10 μg/mL, T100024, Aladdin) was spiked into all samples and calibration standards as the internal standard prior to injection. Calibration curves were constructed for each analyte by plotting Y, defined as the peak area ratio of the analyte to the internal standard, against X, defined as the concentration ratio of the analyte to the internal standard. All calibration curves exhibited excellent linearity across the validated concentration range, with correlation coefficients (*R* ^2^) of at least 0.99 for all compounds.

To evaluate the precision and batch‐to‐batch consistency of the analytical method, three independently prepared batches of samples were analyzed in replicate. The relative standard deviation (RSD) was calculated to assess repeatability. Low RSD values (below 5.0%) for the major components indicated that the method was precise and robust, ensuring the reliability of the quantitative results across different batches.

### 2.3 Animals Model Establishment and Drugs Administration

All animal procedures were ethically approved by the Ethics Committee of the School of Life Sciences, Lanzhou University (Approval ID: EAF2022097). Male Kunming (KM) mice aged 6–8 weeks were obtained from the Lanzhou Veterinary Research Institute, Chinese Academy of Agricultural Sciences (license number: SCXK (GS) 202‐0002) and housed under specific pathogen‐free (SPF) conditions. After a 1‐week acclimatization period, the mice were randomly assigned to six groups using a random number table: control (Con), stressed, fluoxetine‐treated (Flu; 10 mg/kg), KREO‐L (0.0005 mL/kg, equivalent to 0.4225 mg/kg), KREO‐M (0.005 mL/kg, equivalent to 4.225 mg/kg), and KREO‐H (0.05 mL/kg, equivalent to 42.25 mg/kg).^1^ Except for the control group, all other groups were subjected to CUMS to induce depressive‐like behaviors over a 7‐week period (Tian et al. 2020). Standard stressors included 24‐h food and water deprivation, day‐night reversal, crowding, exposure to foreign objects, 5 min of warm‐water swimming (45°C), 5 min of cold‐water swimming (4°C–8°C), body restraint, 2 min of tail pinch, and damp sawdust (Table S1). Drug administration commenced during the third week of the modeling phase, with stressors applied 1 h after dosing. Behavioral tests were conducted on Day 50.

The KREO‐L, KREO‐M, and KREO‐H groups received daily oral gavage with KREO at the respective doses, while the Flu group was administered fluoxetine hydrochloride (Lot No. J20170022, Eli Lilly and Company, China) at 10 mg/kg. The control and stressed groups were treated with an equivalent volume of 0.9% saline solution. The treatment regimen lasted for 35 days.

### 2.4 Animal Behavioral Experiments

#### 2.4.1 Body Weight Measurement

The initial body weights of the mice were recorded to establish a baseline. Thereafter, body weights were monitored weekly throughout the experimental period to evaluate changes in body weight trends across groups.

#### 2.4.2 Forced Swim Test (FST)

Mice were acclimated to the behavioral testing apparatus for 3 h in a temperature‐controlled environment maintained at 20°C–25°C. The test was conducted in a transparent plexiglass cylinder (30 cm high × 18 cm diameter) filled with 18 cm of fresh water maintained at 23°C ± 2°C, ensuring that the mice could not touch the bottom with their hind limbs. Mouse behavior was recorded by video camera for 6 min, and immobility duration was quantified during the last 4 min.

#### 2.4.3 Tail Suspension Test (TST)

Mice were acclimated to the laboratory environment for 3 h prior to testing. During the test, the environment was maintained under quiet and dimly lit conditions, with ambient noise levels below 60 dB and illumination intensity below 100 lx. Each mouse was suspended by the tail from a horizontal rod positioned 50 cm above the ground. The experiment lasted for 6 min, and behavioral activity was recorded during the final 4 min.

#### 2.4.4 Open Field Test (OFT)

The OFT was conducted in a quiet and dimly lit environment. The apparatus consisted of a black plastic enclosure measuring 50 cm × 50 cm × 50 cm. Each mouse was individually placed in the centre of the arena and allowed to freely explore for 5 min. Animal movements were recorded by an overhead video camera and analyzed using SuperMaze software (Shanghai Xinruan Information Technology Co. Ltd., Shanghai, China). The system was used to measure total distance traveled and time spent in the central zone compared to the peripheral zones of the chamber.

### 2.5 Collection of Serum and Hippocampus

Mice were euthanized immediately by cervical dislocation following the completion of behavioral tests. Serum was collected by centrifugation of blood samples at 3000 rpm for 15 min at 4°C and stored at −80°C until further analysis. Hippocampi were homogenized in an appropriate lysis buffer and stored at −80°C for subsequent analysis.

### 2.6 ELISA

5‐HT and BDNF levels in the hippocampus were measured using commercial mouse ELISA kits (5‐HT: MM‐0443M1, BDNF: MM‐0204M1, MEIMIAN, China). Serum corticosterone was assessed using a mouse‐specific ELISA kit (MM‐0061M1, MEIMIAN, China).

### 2.7 WB

The expression levels of TrkB (1:2000, A2099, ABclonal), ERK1/2 (extracellular signal‐regulated kinase 1/2, 1:1000, 11,257–1‐AP, Proteintech), p‐ERK1/2 (1:1000, YP0101, Immunoway), and GAPDH (glyceraldehyde‐3‐phosphate dehydrogenase, 1:1000, 10,494–1‐AP, Proteintech) in mouse hippocampal tissue were determined by WB. Tissues were homogenized (JXFSTPRP, Jingxin, China) in RIPA buffer with 1 mM protease and phosphatase inhibitors at 4°C, centrifuged at 12,000 rpm for 30 min, and supernatants collected for protein quantification (BCA kit, PC002, Solarbio, China). Proteins (20 μg/lane) were separated by SDS‐PAGE (80 V for 30 min, then 120 V for 1 h) and transferred to 0.45 μm PVDF membranes. Membranes were blocked with 5% non‐fat milk in TBST (pH 7.4) for 1 h at room temperature, incubated overnight at 4°C with primary antibodies, washed three times with TBST, then incubated with HRP‐conjugated secondary antibodies for 1 h. After further washes, signals were detected using ECL and quantified by ImageJ, with control group levels normalized to 1.

### 2.8 Statistical Analysis

All data are expressed as mean ± standard error of the mean (SEM) and were analyzed statistically using SPSS 19.0 software. Group differences were assessed by one‐way analysis of variance (ANOVA), followed by Tukey's post hoc test for multiple comparisons. All behavioral assessments and Western blot analyses were performed under blinded conditions to avoid observer bias.

## 3 Results

### 3.1 Chemical Composition of KREO

**FIGURE 1:**
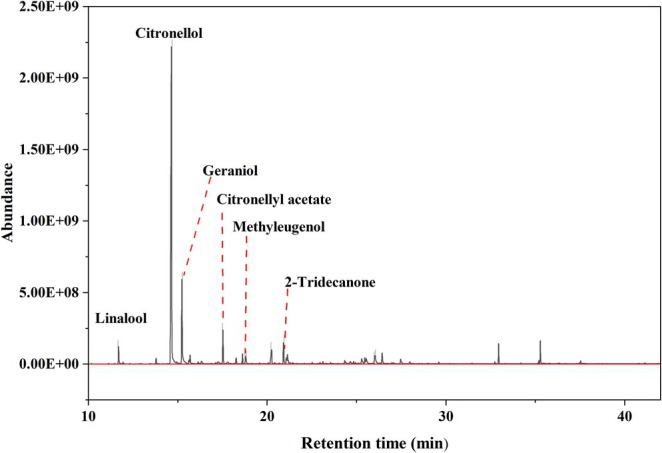
GC–MS chromatogram of KREO.

The GC–MS analysis identified a total of 57 volatile compounds in KREO (refer to Figure 1 and Table S2). As detailed in Table 1, citronellol (312.83 mg/mL) was the predominant constituent, followed by geraniol (73.57 mg/mL) and citronellyl acetate (19.86 mg/mL). These three major components collectively accounted for 69.33% of the total composition. The validation parameters for the quantitative method are provided in Table S3.

**TABLE 1:** Qualitative and quantitative analysis of major compounds in KREO.

	Compound name‐no	Standard retention time (min)	Sample retention time (min)	Area % (relative content)	Quantitative concentration (mg/mL)	RSD (%) (*n* = 3)	Chemical structure
1	Linalool	11.69	11.71	2.40	9.98	6.36%	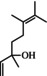
2	Citronellol	14.65	14.63	54.41	312.83	0.26%	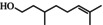
3	Geraniol	15.23	15.31	10.41	73.57	1.98%	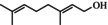
4	Citronellyl acetate	17.53	17.55	4.51	19.86	2.57%	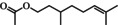
5	Methyleugenol	18.26	18.28	1.97	9.82	3.65%	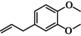
6	2‐Tridecanone	18.79	18.83	3.31	12.90	—	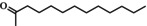

### 3.2 KREO Alleviated Depressive‐Like Behaviors in CUMS Mice

**FIGURE 2:**
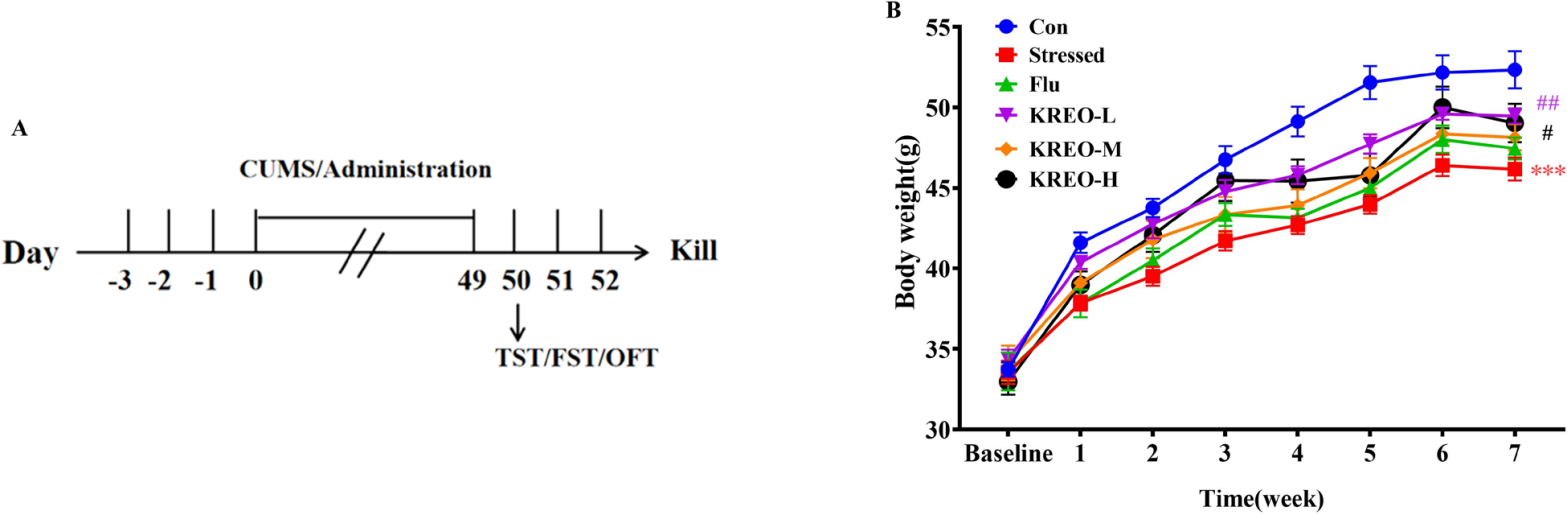
KREO could maintain normal growth in body weight in CUMS‐induced mice. (A) Design of the experiment. (B) Changes in body weight during treatment (*n* = 8). ***p < 0.001, compared with control group; ^#^ *p* < 0.05, ^##^ *p* < 0.01, compared with stressed group.

The experimental timeline is shown in Figure 2A, with body weight measured weekly. As shown in Figure 2B, the body weight of stressed mice increased to a lesser extent than that of the control group. Notably, mice treated with KREO exhibited higher body weight gain compared to the stressed group, with the KREO‐L, KREO‐M, and KREO‐H groups showing recovery rates of 9.24%, 5.15%, and 7.28%, respectively. In contrast, no significant change in body weight was observed in the Flu‐treated group relative to the stressed group.

**FIGURE 3:**
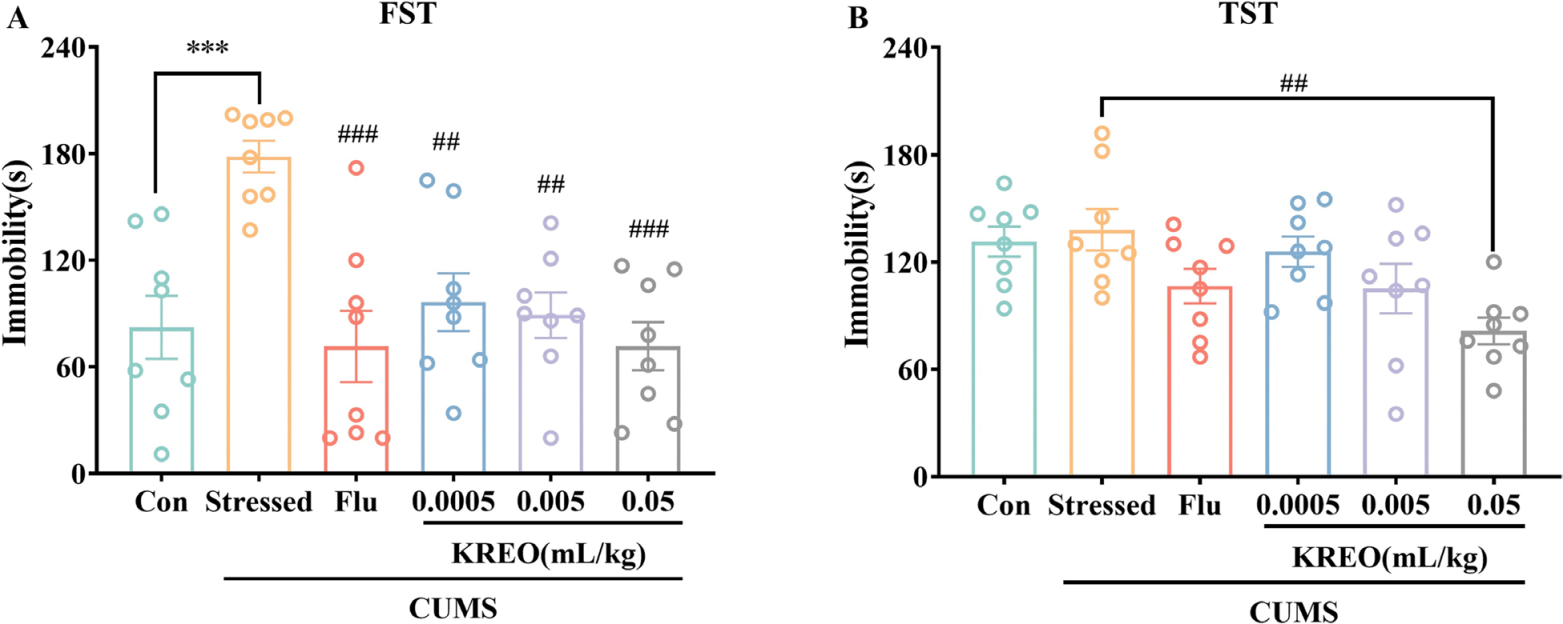
KREO reduced the immobility time in CUMS‐induced mice during the FST and TST. (A) Floating immobility time in FST (*n* = 8). (B) Swing immobility time in TST (*n* = 8). ***p < 0.001, compared with control group; ^##^ *p* < 0.01, ^###^ *p* < 0.001, compared with stressed group.

To evaluate whether KREO ameliorates CUMS‐induced depressive‐like behaviors, behavioral tests including FST and TST were conducted. The effects of KREO and fluoxetine on immobility time are presented in Figure 3. In the FST, the stressed group exhibited significantly longer immobility time (178.38 ± 8.97 s) compared to the control group (82.25 ± 17.72 s). Treatment with fluoxetine (71.50 ± 20.03 s) or KREO (KREO‐L: 96.50 ± 16.30 s; KREO‐M: 89.13 ± 12.78 s; KREO‐H: 71.63 ± 13.53 s) significantly reduced immobility time. Notably, KREO exerted a dose‐dependent effect, with the KREO‐H group showing efficacy comparable to fluoxetine, reducing immobility by 59.85% and 59.92%, respectively. Similarly, in the TST, all KREO‐treated groups displayed decreased immobility, with the KREO‐H group (81.50 ± 7.48 s) exhibiting the most pronounced reduction—40.94% lower than the stressed group (138.00 ± 11.73 s).

**FIGURE 4:**
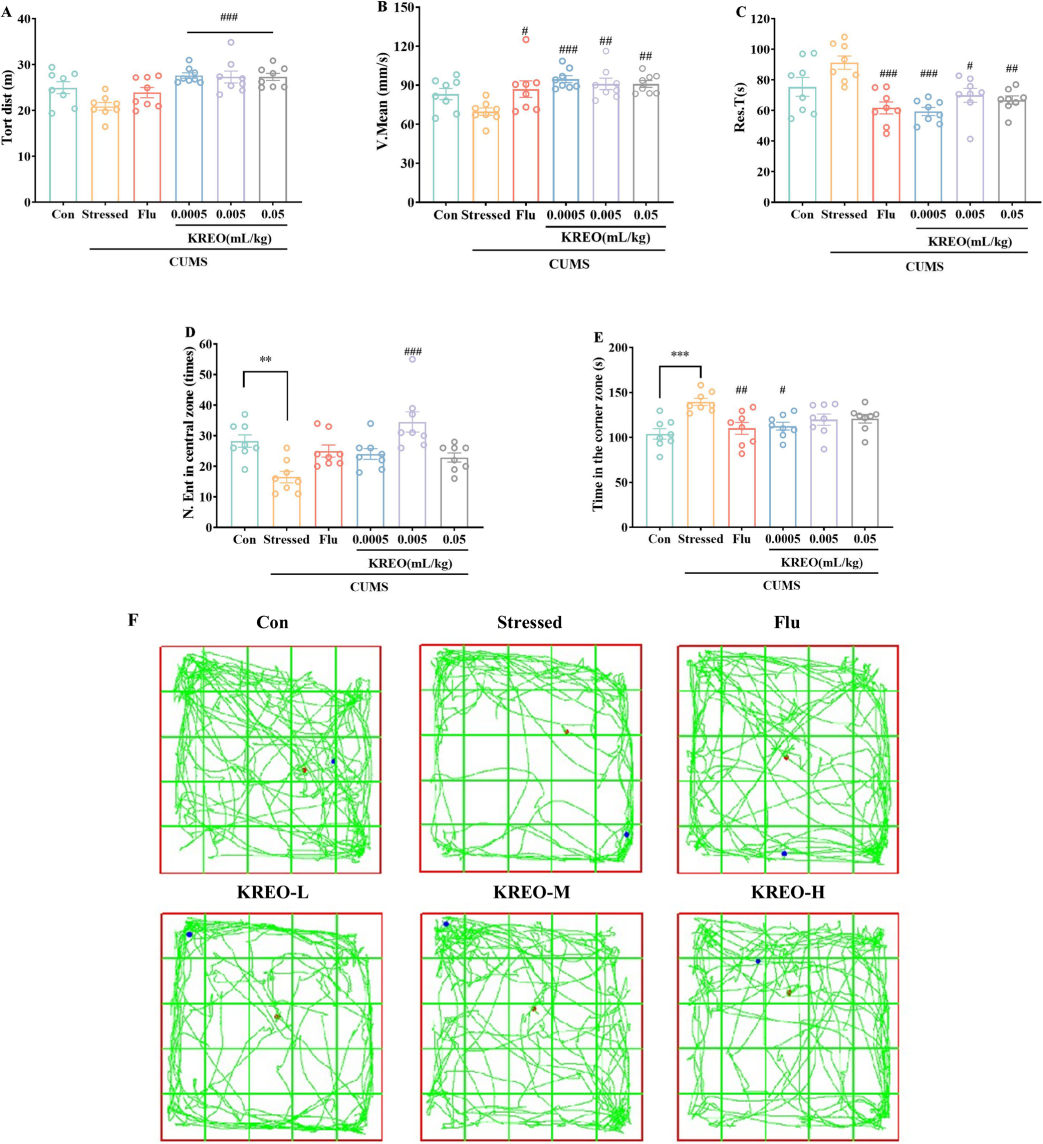
KREO enhanced voluntary activity and exploratory behavior in CUMS mice. (A) Total distance; (B) average velocity; (C) rest time; (D) number of entries in center zone; (E) time in the corner zone; (F) the representative trajectories of mice in different groups in the OFT (*n* = 8). **p < 0.01, ***p < 0.001, compared with control group; ^#^ *p* < 0.05, ^##^ *p* < 0.01, ^###^ *p* < 0.001, compared with stressed group.

To evaluate the effects of KREO on voluntary locomotor activity and exploratory behavior in mice, OFT was performed. Representative movement trajectories and quantitative results are presented in Figure 4. Stressed mice exhibited a significant reduction in central zone entries and increased time spent in peripheral zones compared to controls, indicating impaired spontaneous locomotion and a depressive‐like phenotype. Notably, KREO treatment reversed these behavioral deficits, as evidenced by increased total distance traveled, higher average speed, reduced resting time, greater number of central zone entries, and decreased time spent in corner zones relative to the stressed group. These findings suggest that KREO ameliorates the adverse effects of chronic stress on locomotor and exploratory behaviors in mice.

### 3.3 KREO Reduced Corticosterone and Increased Hippocampal 5‐HT and BDNF Levels

**FIGURE 5:**
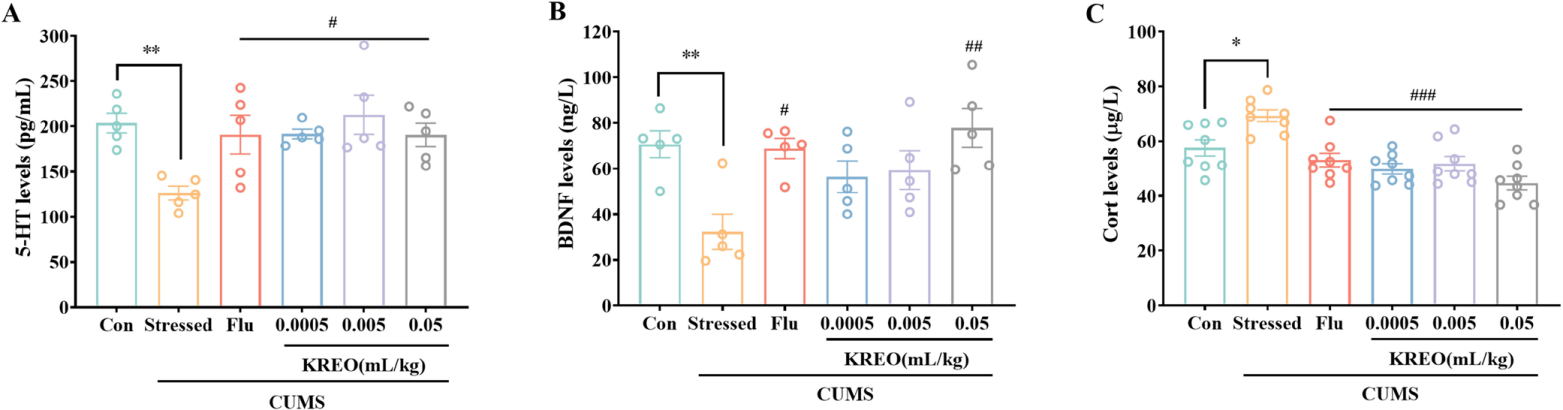
Effect of KREO on the 5‐HT and BDNF level in hippocampus and corticosterone level in serum of CUMS mice. (A) 5‐HT level in hippocampus (*n* = 5). (B) BDNF level in hippocampus (*n* = 5). (C) Corticosterone level in serum (*n* = 8). *p < 0.05, **p < 0.01, compared with control group; ^#^ *p* < 0.05, ^##^ *p* < 0.01, ^###^ *p* < 0.001, compared with stressed group.

Following the completion of behavioral tests, hippocampal tissue and serum samples were collected from mice to measure levels of 5‐HT, BDNF, and corticosterone. 5‐HT modulates central neuronal activity and regulates various physiological functions, including mood, memory, and sleep (Meneses 1998; Sanger 2008). In the stressed group, hippocampal 5‐HT levels were significantly reduced compared to the control group (209.68 ± 10.91 vs. 126.32 ± 7.68 pg/mL, *p* < 0.01), consistent with the characteristic serotonergic deficiency observed in this depression model. After a 5‐week treatment period, 5‐HT levels in the hippocampus increased by 51.68% (KREO‐L: 191.60 ± 5.27 pg/mL), 68.56% (KREO‐M: 212.92 ± 21.75 pg/mL), and 50.86% (KREO‐H: 190.57 ± 12.99 pg/mL) relative to the stressed group (*p* < 0.05; Figure 5A). These results indicate that KREO effectively restores 5‐HT levels across all dose ranges tested, with efficacy comparable to that of fluoxetine treatment.

BDNF, a key neurotrophic factor involved in neuroplasticity and neurotransmitter regulation, is frequently reduced in patients with depression (Boku et al. 2018; Neto et al. 2011). In this study, hippocampal BDNF levels in the stressed group were 54.28% lower than those in the control group (70.68 ± 5.85 vs. 32.32 ± 7.76 ng/L, *p* < 0.01). Treatment with KREO increased BDNF expression, with significantly higher levels observed in both the Flu group (68.82 ± 4.43 ng/L, *p* < 0.05) and the KREO‐H group (77.82 ± 8.56 ng/L, *p* < 0.01) relative to the stressed group, representing increases of 112.94% and 140.79%, respectively (Figure 5B).

In addition, corticosterone—a well‐established biomarker of depression—has been shown to exert neurotoxic effects. As shown in Figure 5C, serum corticosterone levels were significantly elevated in the stressed group compared to the control group (57.52 ± 2.98 vs. 69.29 ± 2.15 μg/L, *p* < 0.05), indicating hyperactivity of the HPA axis in chronically stressed mice. Relative to the stressed group, corticosterone levels were reduced in the Flu (53.07 ± 2.48 μg/L), KREO‐L (52.31 ± 2.31 μg/L), KREO‐M (51.75 ± 2.66 μg/L), and KREO‐H (44.70 ± 2.48 μg/L) groups by 23.42%, 24.51%, 25.32%, and 35.50%, respectively (*p* < 0.001).

These findings indicated that KREO prevented depression‐like behavior by rectifying chronic stress‐induced 5‐HT deficiency, restoring reduced BDNF levels, and normalizing HPA axis hyperactivity.

### 3.4 KREO Regulated Hippocampal Neuroplasticity‐Related Proteins

**FIGURE 6:**
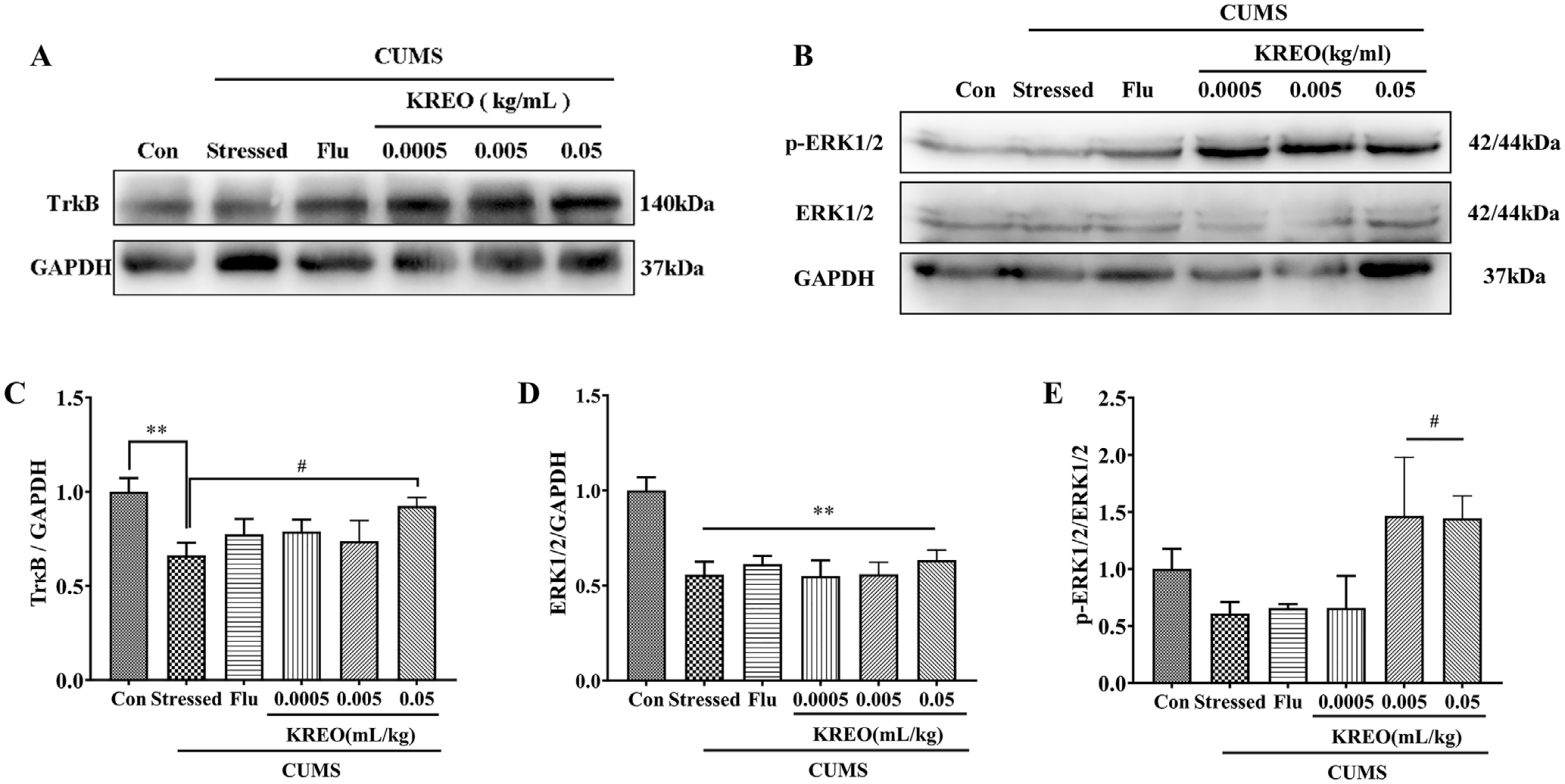
KREO activated the BDNF–TrkB–ERK signaling pathway in the hippocampus of CUMS mice. (A, B) Representative western blots for TrkB, p‐ERK1/2, and ERK1/2 protein expressions in hippocampus tissue extracts. (C) The expression of TrkB in the hippocampus of CUMS mice (*n* = 3). (D, E) The expression of ERK1/2 and p‐ERK1/2 in the hippocampus of CUMS mice (*n* = 3). **p < 0.01, compared with control group; ^#^ *p* < 0.05, compared with stressed group.

To further validate the antidepressant efficacy of KREO through neuroprotective mechanisms, we examined the protein expression in the BDNF signaling pathway within mouse hippocampal tissue. Representative Western blots of TrkB, p‐ERK1/2, and ERK1/2 protein expressions in hippocampal tissue extracts are shown in Figure 6A,B. TrkB, a high‐affinity receptor for BDNF, mediates downstream signaling pathways that are essential for neuronal growth and survival. Antidepressants have been shown to directly activate TrkB pathways (Casarotto et al. 2021). As shown in Figure 6C, the expression of TrkB in the hippocampal tissue of stressed mice was significantly reduced compared to the control group (*p* < 0.01). In contrast, treatment with KREO‐H effectively reversed this downregulation, restoring TrkB protein levels to near‐normal ranges in stressed mice (*p* < 0.05). ERK1/2, a core downstream effector of the BDNF–TrkB pathway, mediates synaptic plasticity, neuronal survival, and antidepressant effects. ERK1/2 and its phosphorylation level were investigated in the hippocampus of mice across experimental groups. Compared with the control group, the stressed group showed a significant reduction in ERK1/2 protein levels in the hippocampal tissue (*p* < 0.01, Figure 6D). Although total ERK1/2 levels were not restored by KREO treatment, the phosphorylation of ERK1/2 was significantly increased following administration of KREO‐M and KREO‐H (*p* < 0.05, Figure 6E).

## 4 Discussion

Dietary interventions serve not only as a therapeutic strategy but also as a form of lifestyle modification capable of influencing the onset and progression of depression through multiple biological mechanisms (Marx et al. 2021). Omega‐3 fatty acids, predominantly sourced from marine foods such as salmon, have been shown to delay the onset of inflammation‐related depressive symptoms (Borsini et al. 2021). Polyphenols present in blueberries and cocoa contribute to brain health by reducing oxidative stress and modulating the gut microbiota composition (Behl et al. 2022; Hao et al. 2023). Plant‐derived essential oils, widely utilized as food additives, not only enhance flavor but also confer potential nutritional and health‐promoting benefits. For example, garlic essential oil, with diallyl disulfide as its primary bioactive component, has been shown to alleviate depressive‐like behaviors in CUMS‐induced rats by modulating the nucleotide‐binding oligomerization domain‐like receptor protein 3 inflammasome pathway in the brain, enhancing intestinal barrier integrity, and restoring gut microbiota balance (Huang et al. 2023). Oral administration of virgin coconut oil in chronic restraint stress rats reduces brain lipid peroxidation, alleviates oxidative damage, and improves spatial learning, memory, and neuronal function, supporting nervous system homeostasis (Dafale et al. 2025). Chinese Kushui Rose, a unique species native to Gansu Province in northwest China, is renowned for its sweet flavor, fragrant aroma, and extensive use in a variety of food products. Despite its commercial value, research on Chinese Kushui Rose remains relatively limited compared to other rose varieties, particularly concerning the therapeutic potential of its bioactive compounds. One such compound is KREO, which prior studies have demonstrated to extend sleep duration in murine models (Luo et al. 2018). Ding et al. (2019) compared the anxiolytic effects of KREO with those of *Rosa damascena* essential oil in the Elevated Plus Maze and Light–Dark Transition tests, demonstrating that KREO exhibits superior anxiolytic efficacy. In the transgenic *C. elegans* model, KREO was shown to significantly suppress Aβ deposition and reduce levels of Aβ oligomers, thereby alleviating toxicity caused by Aβ overexpression (Zhu et al. 2017). KREO was shown to reduce the formation of α‐Syn aggregates and attenuate dopaminergic neuron loss, thereby exerting anti‐Parkinson's effects through the mitigation of oxidative stress via activation of the antioxidant pathway (Muhammad et al. 2023). Furthermore, the two primary bioactive constituents of KREO—citronellol and geraniol—demonstrate robust antidepressant‐like effects in murine models in previous reports. Specifically, *β‐*citronellol has been shown to reverse deficits induced by chronic restraint stress (CRS), effectively increasing locomotor activity in the OFT and reducing immobility in FST (Zhuang et al. 2022). Similarly, geraniol counteracts anhedonia and behavioral despair in the CUMS model by restoring sucrose preference and decreasing immobility in both FST and TST (Deng et al. 2015). Notably, the concentrations of these two key components in KREO are higher than those found in the widely accepted *Rosa damascena* essential oil (Ding et al. 2019). This elevated content may constitute the critical biochemical foundation underpinning KREO's superior antidepressant efficacy. Building on this evidence, the present study aims to evaluate the ability of KREO to prevent depressive‐like behavior and to explore the underlying mechanisms.

In this study, the CUMS model was employed to evaluate the preventive effects of KREO against depression‐like behaviors. This model replicates key symptoms of depression by exposing animals to a series of unpredictable and mild stressors, thereby reflecting the behavioral and neurobiological characteristics associated with human depressive states (Willner 2017). The depressive‐like state in animals was evaluated by monitoring changes in body weight and conducting a battery of behavioral assays, including TST, FST, and OFT. Results from these behavioral assays demonstrated that KREO administration significantly alleviated CUMS‐induced depressive‐like behaviors. Specifically, KREO treatment led to improved body weight recovery, reduced immobility durations in both the TST and FST, and increased locomotor activity in the OFT. Notably, the high dose of KREO exhibited the most robust therapeutic response, demonstrating a 59.85% reduction in immobility time in the FST and a 40.94% decrease in immobility time in the TST.

Stress‐induced hormonal and neuroendocrine dysfunction have been shown to play a significant role in the pathophysiology of depression. The HPA axis, a central neuroendocrine system responsible for coordinating the stress response, is activated under stressful conditions (Dwyer et al. 2020). Activation of the HPA axis stimulates the adrenal cortex to increase secretion of glucocorticoids—cortisol in humans and corticosterone in rodents—which regulate various physiological processes (Willner et al. 2013). The amygdala exerts a positive influence on the HPA axis, leading to elevated cortisol levels, whereas the hippocampus provides negative feedback modulation, thereby maintaining homeostatic control over glucocorticoid levels (Jacobson and Sapolsky 1991; Vyas et al. 2002). These two brain regions act in concert to regulate the physiological activity of the HPA axis. This hypercortisolemia can exert detrimental effects on hippocampal granule cells, primarily through the downregulation of glucocorticoid receptors and subsequent granule cell atrophy (Hartmann et al. 2021). The consequences of impaired GR‐mediated signaling in these cells are substantial and include reduced 5‐HT levels in the synaptic cleft due to increased monoamine oxidase‐A activity, enhanced activation of calcium‐dependent enzymes leading to the production of neurotoxic free radicals, and diminished BDNF expression (Magariños et al. 1996; McEwen 2005; Meyer 2012). 5‐HT is a crucial neurotransmitter in the central nervous system (CNS), critically involved in regulating key physiological and psychological functions, including mood, sleep, and appetite. Elevated 5‐HT levels in the CNS are associated with enhanced emotional well‐being and reduced anxiety, underpinning the pharmacological mechanism of many widely prescribed antidepressant medications (Nedic Erjavec et al. 2021). Furthermore, upregulation of BDNF enhances neuroplasticity and promotes neurogenesis, thereby contributing to a sustained antidepressant effect (Boku et al. 2018; Harmer et al. 2017). In our study, KREO administration effectively reversed stress‐induced alterations in hippocampal 5‐HT and BDNF levels as well as serum corticosterone concentrations in CUMS‐exposed mice. After a 5‐week treatment period, hippocampal 5‐HT levels increased by 51.68%, 68.56%, and 50.86% in the KREO‐L, KREO‐M, and KREO‐H groups, respectively, relative to the stressed group. Notably, high‐dose KREO significantly enhanced hippocampal BDNF expression by 140.79%. Serum corticosterone levels were normalized across all treatment groups, with the high‐dose KREO group exhibiting a 35.50% reduction compared to the stressed group. Collectively, these findings demonstrate that KREO ameliorates 5‐HT deficits, restores BDNF signaling, and attenuates HPA axis hyperactivity in the hippocampus, exerting regulatory effects comparable to those of conventional antidepressants. These findings demonstrate that KREO exerts a preventive effect against depression‐like behaviors by correcting chronic stress‐induced 5‐HT deficiency, restoring reduced BDNF levels, and normalizing HPA axis dysfunction in a murine model of depression.

To further investigate the preventive potential of KREO against depression‐like behaviors through neuroprotective mechanisms, we examined protein expression in the BDNF signaling pathway within mouse hippocampal tissue. Previous studies have established the BDNF–TrkB axis as a fundamental pathway underlying antidepressant efficacy (Luo et al. 2014; Sheline et al. 1996). The signaling cascade is initiated when antidepressants promote BDNF binding to TrkB, triggering ERK1/2—a core downstream effector of the BDNF–TrkB pathway—to transduce neurotrophic signals from the cell surface to the nucleus, thereby regulating gene expression and mediating synaptic plasticity, neuronal survival, and antidepressant effects (Wang and Mao 2019). This signaling ultimately leads to the modulation and activation of key neurotransmitter receptors, including 5‐HT, adrenergic, dopamine, and glutamate receptors (Casarotto et al. 2021). For example, ketamine produces rapid antidepressant effects primarily by blocking NMDARs and activating the downstream BDNF–TrkB–ERK signaling cascade (Li et al. 2019; Ma et al. 2017); however, its clinical utility remains limited by high abuse liability and addictive potential (Rolfzen et al. 2024). In contrast, KREO—a natural, edible essential oil—effectively modulates this key signaling pathway to exert antidepressant‐like effects. In our study, KREO intervention restored TrkB and p‐ERK1/2 levels in the CUMS model, with the most pronounced effects observed in the KREO‐H group. These findings suggest a potential association between KREO's preventive effects against depression‐like behaviors and the BDNF–TrkB–ERK signaling pathway—characterized by increased BDNF expression and secretion, TrkB activation, and downstream signaling. This modulation may underlie the observed improvements in synaptic plasticity and neurogenesis, pointing to a plausible mechanism of action in preventive intervention.

This study represents the first systematic assessment of KREO's preventive efficacy against depression‐like behaviors and provides preliminary insights into its mechanisms of action. Our study has limitations in behavioral scope and mechanistic depth. Future work will integrate metabolomics and chromatography to identify active constituents, expand behavioral phenotyping with the sucrose preference test, and include female mice to assess sex differences. Concurrently, we are applying targeted interventions—including selective TrkB antagonism, region‐specific knockdown in the prefrontal cortex and hippocampus, and vagotomy—to definitively establish causal mechanisms and validate the role of the gut‐microbiota‐brain axis.

## 5 Conclusions

This study demonstrates that KREO effectively ameliorates CUMS‐induced depressive‐like behaviors in mice. Mechanistically, KREO enhances monoaminergic neurotransmission, suppresses HPA axis hyperactivity, and is associated with activation of the BDNF signaling pathway, collectively coinciding with improved synaptic plasticity and neural regeneration. These findings support the potential of KREO as a promising candidate for the development of novel therapeutics in the treatment of depression.

## Author Contributions

**Manjie Xu:** data curation, validation. **Yanni Wang:** data curation, formal analysis, investigation, writing – original draft. **Kailong Guan:** formal analysis. **Wenjun Xu:** investigation, data curation. **Xiaolong Li:** formal analysis. **Zewen Zhang:** validation. **Qinghong Liu:** validation. **Weizhong Zhang:** formal analysis. **Xin Wang:** conceptualization, supervision, writing – review and editing. **Zhaorong Yue:** formal analysis, investigation. **Yang Li:** conceptualization, writing – review and editing. **Hongyu Li:** conceptualization, supervision, writing – review and editing.

## Funding

This work was supported by the Science and Technology Program of Gansu Province—Special Project of the Science and Technology Innovation Fund for Small and Medium‐sized Enterprises (“Innovation and Development of Rose Gel Candies”, Grant No. 24CXGA097) and the Science and Technology Department of Gansu Province (“Innovation and Development of Rose Chewing Gum Series Products”).

## Ethics Statement

This study was ethically approved by the Ethics Committee of the School of Life Sciences, Lanzhou University (Approval ID: EAF2022097).

## Conflicts of Interest

The authors declare no conflicts of interest.

## Supporting information


**Table S1:** Schedule of CUMS stimulation.
**Table S2:** Chemical composition of volatile compounds in KREO.
**Table S3:** Method validation parameters for quantitative analysis.

## Data Availability

The data that support the findings of this study are available from the corresponding author upon reasonable request.

## Endnotes

^1^

*Note: The conversion is based on the density of KREO (1 mL = 0.845 g)*.
