# Supplementary material for: KREO Prevents CUMS‐Induced Depressive‐Like Behavior via Modulation of the BDNF Signaling Pathway
Source: Food Sci Nutr. 2026 May 27;14(6):e71912. doi: 10.1002/fsn3.71912 (PMC13238901; doi:10.1002/fsn3.71912)
Supplement: Supplementary file 1 — Table S1: Schedule of CUMS stimulation. Table S2: Chemical composition of volatile compounds in KREO. Table S3: Method validation parameters for quantitative analysis. [file FSN3-14-e71912-s001.docx]

# Section S1

Table S1: Schedule of CUMS stimulation

| Week | Monday | Tuesday | Wednesday | Thursday | Friday | Saturday | Sunday |
| --- | --- | --- | --- | --- | --- | --- | --- |
| **1** | Food deprivation | Damp sawdust | Day-night reversal | Crowding | Water deprivation | Foreign object stimulation | Warm-water swimming |
| **2** | Cold-water swimming | Water deprivation | Body restriction | Day-night reversal | Tail pinching | Damp sawdust | Crowding |
| **3** | Water deprivation | Foreign object stimulation | Warm-water swimming | Food deprivation | Body restriction | Day-night reversal | Tail pinching |
| **4** | Damp sawdust | Warm-water swimming | Water deprivation | Crowding | Cold-water swimming | Food deprivation | Body restriction |
| **5** | Day-night reversal | Tail pinching | Damp sawdust | Foreign object stimulation | Water deprivation | Warm-water swimming | Cold-water swimming |
| **6** | Tail pinching | Foreign object stimulation | Damp sawdust | Crowding | Water deprivation | Warm-water swimming | Body restriction |
| **7** | Food deprivation | Body restriction | Day-night reversal | Tail pinching | Damp sawdust | Crowding | Water deprivation |

# Section S2

Table S2: Chemical Composition of Volatile Compounds in KREO

| No | Compound | Molecular formula | CAS | Retention time  (min) | Similarity Index (SI) | Content  (%) |
| --- | --- | --- | --- | --- | --- | --- |
| 1 | cis-Linalool oxide | C_10_H_18_O_2_ | 5989-33-3 | 11.13 | 917 | 0.08 |
| 2 | trans-Linalool oxide | C_10_H_18_O_2_ | 34995-77-2 | 11.47 | 904 | 0.07 |
| 3 | Linalool | C_10_H_18_O | 78-70-6 | 11.69 | 970 | 2.40 |
| 4 | (-)-cis-Rose oxide | C_10_H_18_O | 3033-23-6 | 11.93 | 916 | 0.16 |
| 5 | trans-Rose oxide | C_10_H_18_O | 876-18-6 | 12.31 | 915 | 0.07 |
| 6 | 2,6,6-Trimethyl-2-cyclohexene-1-methanol | C_10_H_18_O | 6627-74-3 | 13.45 | 809 | 0.05 |
| 7 | alpha-Terpineol | C_10_H_18_O | 98-55-5 | 13.79 | 945 | 0.69 |
| 8 | Citronellol | C_10_H_20_O | 106-22-9 | 14.64 | 942 | 54.41 |
| 9 | Neral | C_10_H_16_O | 106-26-3 | 14.93 | 877 | 0.12 |
| 10 | Geraniol | C_10_H_18_O | 106-24-1 | 15.23 | 945 | 10.41 |
| 11 | Geranial | C_10_H_16_O | 141-27-5 | 15.62 | 938 | 0.33 |
| 12 | Citronellyl formate | C_11_H_20_O_2_ | 105-85-1 | 15.69 | 914 | 0.97 |
| 13 | α-Bergamotene | C_15_H_24_ | 17699-05-7 | 15.97 | 930 | 0.12 |
| 14 | 2-Undecanone | C_11_H_22_O | 112-12-9 | 16.14 | 950 | 0.26 |
| 15 | 2-Undecanol | C_11_H_24_O | 1653-30-1 | 16.33 | 887 | 0.46 |
| 16 | Citronellyl acetate | C_12_H_22_O_2_ | 150-84-5 | 17.53 | 945 | 4.51 |
| 17 | Eugenol | C_10_H_12_O_2_ | 97-53-0 | 17.75 | 905 | 0.18 |
| 18 | Bicyclogermacrene | C_15_H_24_ | 24703-35-3 | 18.14 | 821 | 0.07 |
| 19 | Geranyl acetate | C_12_H_20_O_2_ | 105-87-3 | 18.26 | 822 | 0.81 |
| 20 | Tetradecane | C_14_H_30_ | 629-59-4 | 18.62 | 959 | 1.10 |
| 21 | Methyl eugenol | C_11_H_14_O_2_ | 93-15-2 | 18.79 | 915 | 1.97 |
| 22 | (+)-Aromadendrene | C_15_H_24_ | 489-39-4 | 20.24 | 943 | 3.18 |
| 23 | (-)-alpha-Copaene | C_15_H_24_ | 3856-25-5 | 20.42 | 860 | 0.18 |
| 24 | α-Curcumene | C_15_H_22_ | 644-30-4 | 20.67 | 926 | 0.13 |
| 25 | 2-Tridecanone | C_13_H_26_O | 593-08-8 | 20.91 | 945 | 3.31 |
| 26 | 2-Tridecanol | C_13_H_28_O | 1653-31-2 | 21.07 | 925 | 0.66 |
| 27 | Isodaucene | C_15_H_24_ | 142878-08-8 | 21.13 | 914 | 1.42 |
| 28 | 2-Methyl-5-(6-methylhept-5-en-2-yl)bicyclo[3.1.0]hexan-2-ol | C_15_H_26_O | 145512-84-1 | 21.27 | 809 | 0.10 |
| 29 | (3R,3aR,3bR,4S,7R,7aR)-octahydro-3,7-dimethyl-4-(1-methylethyl)-1H-cyclopentacyclopropabenzen-3-ol | C_15_H_26_O | 38230-60-3 | 21.47 | 813 | 0.07 |
| 30 | β-Cadinene | C_15_H_24_ | 523-47-7 | 21.65 | 825 | 0.05 |
| 31 | trans-Nerolidol | C_15_H_26_O | 40716-66-3 | 22.52 | 939 | 0.16 |
| 32 | Cubebol | C_15_H_26_O | 23445-02-5 | 22.84 | 812 | 0.07 |
| 33 | (-)-Spathulenol | C_15_H_24_O | 77171-55-2 | 22.95 | 910 | 0.14 |
| 34 | Sotorasib | C_15_H_26_O_2_ | 2117730-73-9 | 23.12 | 836 | 0.27 |
| 35 | Ledol | C_15_H_26_O | 577-27-5 | 23.54 | 831 | 0.20 |
| 36 | Dichloro(1,3-bis(2,4,6-trimethylphenyl)imidazol-2-ylidene)palladium(II) | C_15_H_22_O | 311351-24-3 | 24.34 | 823 | 0.43 |
| 37 | trans-α-Santalol | C_15_H_24_O | 19903-72-1 | 24.41 | 800 | 0.13 |
| 38 | α-Bisabolol oxide B | C_15_H_26_O_2_ | 26184-88-3 | 24.65 | 831 | 0.45 |
| 40 | Elemol | C_15_H_26_O | 639-99-6 | 24.94 | 809 | 0.13 |
| 41 | alpha-Bisabolol | C_15_H_26_O | 515-69-5 | 25.28 | 910 | 0.88 |
| 42 | Geranylgeraniol | C_20_H_34_O | 24034-73-9 | 25.54 | 834 | 0.81 |
| 43 | (E, E)-Farnesol | C_15_H_26_O | 106-28-5 | 26.02 | 909 | 2.37 |
| 44 | T-Cadinol | C_15_H_22_O | 41610-68-8 | 26.43 | 853 | 1.46 |
| 45 | Siponimod | C_15_H_22_O | 352457-39-7 | 27.46 | 839 | 0.76 |
| 46 | Farnesyl acetate | C_17_H_28_O_2_ | 4128-17-0 | 28.48 | 839 | 0.08 |
| 47 | Nonadecane | C_19_H_40_ | 629-92-5 | 29.60 | 925 | 0.24 |
| 48 | Citronellyl benzoate | C_17_H_24_O_2_ | 10482-77-6 | 30.20 | 872 | 0.08 |
| 49 | n-Tetracosane | C_24_H_50_ | 646-31-1 | 31.46 | 897 | 0.08 |
| 50 | n-Heneicosane | C_21_H_44_ | 629-94-7 | 32.94 | 960 | 1.96 |
| 51 | Octadecane | C_18_H_38_ | 593-45-3 | 34.19 | 896 | 0.10 |
| 52 | Hexacos-1-ene | C_26_H_52_ | 18835-33-1 | 35.19 | 937 | 0.25 |
| 54 | Nonacos-1-ene | C_29_H_58_ | 18835-35-3 | 37.45 | 894 | 0.07 |
| 55 | n-Heptacosane | C_27_H_56_ | 593-49-7 | 37.53 | 907 | 0.44 |
| 57 | (-)-Cyclocolorenone | C_24_H_46_O_2_ | 3681-72-9 | 41.12 | 896 | 0.13 |

# Section S3

Table S3. Method Validation Parameters for Quantitative Analysis

| Compound | Linear Range (μg/mL) | Regression Equation | *R*^2^ | RSD (%) (n=3) |
| --- | --- | --- | --- | --- |
| Linalool | 3.45-1723.30 | y = 0.4558x + 0.5244 | 0.9995 | 6.36% |
| Citronellol | 5.04-504.20 | y = 0.4535x + 0.3543 | 0.9923 | 0.26% |
| Geraniol | 7.02-1754.31 | y = 0.7356x + 1.3382 | 0.9986 | 1.98% |
| Citronellyl acetate | 3.49-1746.36 | y = 0.3213x + 0.5655 | 0.9993 | 2.57% |
| Methyleugenol | 4.14-413.53 | y = 0.461x + 0.2268 | 0.9956 | 3.65% |
